# Sentinel Node Procedure to Select Clinically Localized Prostate Cancer Patients with Occult Nodal Metastases for Whole Pelvis Radiotherapy

**DOI:** 10.1016/j.euros.2022.12.011

**Published:** 2023-01-30

**Authors:** Hilda A. de Barros, Jan J. Duin, Daan Mulder, Vincent van der Noort, M. Arjen Noordzij, Esther M.K. Wit, Floris J. Pos, Wouter V. Vogel, Eva E. Schaake, Fijs W.B. van Leeuwen, Pim J. van Leeuwen, Nikolaos Grivas, Henk G. van der Poel

**Affiliations:** a Department of Urology, The Netherlands Cancer Institute-Antoni van Leeuwenhoek Hospital, Amsterdam, The Netherlands; b Prostate Cancer Network the Netherlands, Amsterdam, The Netherlands; c Department of Biometrics, The Netherlands Cancer Institute-Antoni van Leeuwenhoek Hospital, Amsterdam, The Netherlands; d Department of Urology, Spaarne Gasthuis, Hoofddorp, The Netherlands; e Department of Radiation Oncology, The Netherlands Cancer Institute-Antoni van Leeuwenhoek Hospital, Amsterdam, The Netherlands; f Department of Nuclear Medicine, The Netherlands Cancer Institute-Antoni van Leeuwenhoek Hospital, Amsterdam, The Netherlands; g Interventional Molecular Imaging Laboratory, Department of Radiology, Leiden University Medical Center, Leiden, The Netherlands; h Department of Urology, Amsterdam University Medical Center, Location VUmc, Amsterdam, The Netherlands

**Keywords:** Prostate cancer, Sentinel lymph node procedure, Prostate-only radiotherapy, Whole pelvis radiotherapy

## Abstract

**Background:**

Accurate identification of men who harbor nodal metastases is necessary to select patients who most likely benefit from whole pelvis radiotherapy (WPRT). Limited sensitivity of diagnostic imaging approaches for the detection of nodal micrometastases has led to the exploration of the sentinel lymph node biopsy (SLNB).

**Objective:**

To evaluate whether SLNB can be used as a tool to select pathologically node-positive patients who likely benefit from WPRT.

**Design, setting, and participants:**

We included 528 clinically node-negative primary prostate cancer (PCa) patients with an estimated nodal risk of >5% treated between 2007 and 2018.

**Intervention:**

A total of 267 patients were directly treated with prostate-only radiotherapy (PORT; non-SLNB group), while 261 patients underwent SLNB to remove lymph nodes directly draining from the primary tumor prior to radiotherapy (SLNB group); pN0 patients were treated with PORT, while pN1 patients were offered WPRT.

**Outcome measurements and statistical analysis:**

Biochemical recurrence–free survival (BCRFS) and radiological recurrence-free survival (RRFS) were compared using propensity score weighted (PSW) Cox proportional hazard models.

**Results and limitations:**

The median follow-up was 71 mo. Occult nodal metastases were found in 97 (37%) SLNB patients (median metastasis size: 2 mm). Adjusted 7-yr BCRFS rates were 81% (95% confidence interval [CI] 77–86%) in the SLNB group and 49% (95% CI 43–56%) in the non-SLNB group. The corresponding adjusted 7-yr RRFS rates were 83% (95% CI 78–87%) and 52% (95% CI 46–59%), respectively. In the PSW multivariable Cox regression analysis, SLNB was associated with improved BCRFS (hazard ratio [HR] 0.38, 95% CI 0.25–0.59, *p* < 0.001) and RRFS (HR 0.44, 95% CI 0.28–0.69, *p* < 0.001). Limitations include the bias inherent to the study’s retrospective nature.

**Conclusions:**

SLNB-based selection of pN1 PCa patients for WPRT was associated with significantly improved BCRFS and RRFS compared with (conventional) imaging-based PORT.

**Patient summary:**

Sentinel node biopsy can be used to select patients who will benefit from the addition of pelvis radiotherapy. This strategy results in a longer duration of prostate-specific antigen control and a lower risk of radiological recurrence.

## Take Home Message

In clinically localized high-risk prostate cancer patients, sentinel lymph node biopsy–based selection of men with occult nodal metastases for whole pelvis radiotherapy is associated with favorable oncological outcomes as compared with imaging-based prostate-only radiotherapy.

## 1 Introduction

Presence of lymph node (LN) metastases is considered an important prognostic factor in prostate cancer (PCa), as these are associated with a higher likelihood of disease progression and dissemination [1]. Currently, the European Association of Urology guidelines recommend prostate-only radiotherapy (PORT) combined with androgen deprivation therapy (ADT) for clinically node-negative (cN0) PCa patients, regardless of the estimated risk of nodal metastases [2]. Whole pelvis radiotherapy (WPRT) has shown encouraging results in pathologically node-positive PCa patients [3], [4]. Recently, also prophylactic WPRT was proved to provide a significant survival benefit over PORT in cN0, high-risk PCa patients [5]. However, WPRT comes with increased toxicity [6], [7], [8]. Accurate identification of men who in fact harbor nodal metastases is necessary to select patients who are likely to benefit from WPRT. Conventional imaging (ie, computed tomography [CT] or magnetic resonance imaging [MRI]) techniques have insufficient sensitivity to detect nodal (micro)metastases [9]. However, novel molecular imaging approaches (ie, prostate-specific membrane antigen [PSMA] positron emission tomography [PET]/CT) also fail to detect nodal metastases <3 mm [10], [11].

Extended pelvic lymph node dissection (ePLND)— the gold standard for nodal staging in clinically localized PCa—has been used as a staging tool for WPRT [3], [4]. However, ePLND has been associated with increased morbidity, and its template does not include aberrant lymphatic draining sites of the prostate [12], [13]. The ability to identify the location of nodal metastases based on the lymphatic drainage of the primary tumor has led to the exploration of the sentinel lymph node biopsy (SLNB). In PCa surgery, SLNB-directed dissections have yielded a diagnostic accuracy comparable with that of ePLND, but with lower complication rates [14], [15]. Critically, SLNB helps identify aberrant drainage outside the standard ePLND template, which is seen in up to a third of the prostatic sentinel nodes (SNs) [13], [16]. The objective of this study was to evaluate whether SN sampling in cN0 patients with an increased risk of nodal metastases, followed by selection of pN1 patients for WPRT, improved the oncological outcomes as compared with conventional imaging-based PORT.

## 2 Patients and methods

### 2.1 Study design and patient population

This retrospective cohort study included cN0 PCa patients with a >5% Briganti et al [17] 2012 nomogram–assessed risk of lymph node metastasis (LNM) scheduled for external beam radiotherapy in two tertiary referral centers (ie, The Netherlands Cancer Institute [NCI] and Spaarne Gasthuis) between 2007 and 2018. Approval of the institutional review board was obtained before patient identification (IRBdm21-216). Prior to radiotherapy, the majority of patients received nodal staging with conventional imaging (ie, CT and bone scan), and only a minority was staged with PSMA PET/CT imaging. All patients at the NCI received (robot-assisted) laparoscopic SLNB (SLNB group) prior to radiotherapy, and the radiotherapeutic field was based on the histopathological outcome of the SLNB procedure (SLNB-guided radiotherapy). In case of a histologically negative SN (pN0), patients received PORT. Patients with a histologically positive SN (pN1) received WPRT. Patients at the Spaarne Gasthuis did not receive SLNB, but were all offered PORT (non-SLNB group). The exclusion criteria included metastatic disease on imaging at the time of diagnosis, pelvic LN enlargement (LN short-axis diameter ≥10 mm), previous PCa treatment, or World Health Organization performance status ≥3.

### 2.2 SLNB procedure

For the detection of SNs up to 2012, ^99m^Technetium (Tc)-nanocolloid was used as a tracer (*n* = 113), and from 2012 onward, the hybrid tracer indocyanine green (ICG)-^99m^Tc-nanocolloid was used (*n* = 148). The detailed SLNB procedures are described in the Supplementary material*.* SNs were surgically removed using a laparoscopic setup (as described previously [18]) or the da Vinci Si Surgical System (Intuitive Surgical Inc., Sunnyvale, CA, USA). Preoperatively acquired single-photon emission computerized tomography (SPECT)-CT images provided a roadmap for the localization of SNs. Intraoperatively, SNs were first pursued using gamma tracing, followed by fluorescence imaging confirmation. In case of a one-sided nonvisualization of SN on preoperative imaging (*n* = 8), ePLND up to the ureter-vessel crossing was performed ipsilaterally, defined as the removal of nodes from the bifurcation of the common iliac artery up to the ureteral crossing, along the external and internal iliac vessels (with the deep circumflex vein and femoral canal as the distal border) and the obturator fossa (with the genitofemoral nerve as the lateral border). SNs were fixed in formalin, cut into 2 mm segments, embedded in paraffin, and stained with hematoxylin and eosin. An immunohistochemical evaluation was performed with the CAM5.2 monoclonal antibody.

### 2.3 PORT and WPRT procedures

Patients were treated with 75.25–77 Gy to the prostate, and an additional 52.5–56 Gy to pelvic LNs was offered in 35 or 39 fractions in case of a positive SN. Pelvic LN regions were contoured based on the Radiation Therapy Oncology Group (RTOG) guidelines [19]. Treatment was delivered using intensity-modulated radiotherapy until June 2014 and using the volumetric-modulated arc therapy technique thereafter.

### 2.4 Androgen deprivation therapy

Androgen suppression started before the initiation of radiotherapy and consisted of luteinizing hormone-releasing hormone (ant)agonists and/or antiandrogens. Patients with high-risk disease or locally advanced disease were offered 18–36 mo of ADT. Patients with a histologically positive SN were offered 36 mo of ADT. Low- and intermediate-risk PCa patients generally received up to 6 mo of ADT.

### 2.5 Follow-up and outcomes

Prostate-specific antigen (PSA) levels were evaluated every 4 mo during the first 3 yr after radiotherapy and twice a year thereafter. Biochemical recurrence (BCR) was defined as a PSA nadir plus 2 ng/ml in accordance with the Phoenix definition [20]. In case of a BCR or symptomatic disease, imaging was performed for restaging. Patients with a radiological recurrence after treatment received salvage treatment according to the treating physician’s choice. The primary outcome measure was BCR-free survival (BCRFS), defined as the interval between the end of radiotherapy and the occurrence of a BCR. The secondary outcomes were radiological recurrence-free survival (RRFS), defined as the interval between the end of treatment and the diagnosis of any type of recurrence on imaging, and disease-specific survival (DSS), defined as the interval between the end of treatment and PCa-related death. Additional outcomes were 90-d Clavien-Dindo surgical complications after SLNB and treatment-associated toxicities using the Common Terminology Criteria for Adverse Events v5.0 grading system. Only common grade ≥2 toxicities associated with ADT and radiotherapy were recorded.

### 2.6 Statistical analysis

To compare continuous variables between treatment groups (ie, SLNB and non-SLNB groups), an unpaired *T* test or a Mann-Whitney *U* nonparametric test was used. A chi-square test or a Fisher’s exact test was performed to compare discrete variables. Since patients were not randomly assigned to both treatment groups, we performed a propensity score analysis based on the inverse probability of treatment weighting (IPTW) [21]. Propensity scores were generated using a multivariable logistic regression adjusting for the following variables: age, cT stage, log_2_iPSA, International Society of Urological Pathologists (ISUP) grade group, and ADT duration (Supplementary Table 1). To identify factors associated with survival outcomes, propensity score weighted (PSW) Cox proportional hazard regression models were used. The proportional hazard assumption was tested both graphically and with Schoenfeld residuals. Covariate (ie, age, cT stage, log_2_iPSA, ISUP grade group, and ADT duration)-adjusted Kaplan-Meier survival curves were generated for BCRFS, RRFS, and DSS using IPTW. A two-sided *p* value of <0.05 was considered statistically significant. Statistical analyses were performed using both R version 4.0.1 (R Foundation for Statistical Computing, Vienna, Austria) and IBM SPSS Statistics 27 (IBM Inc., Chicago, IL, USA).

## 3 Results

**Fig. 1:**
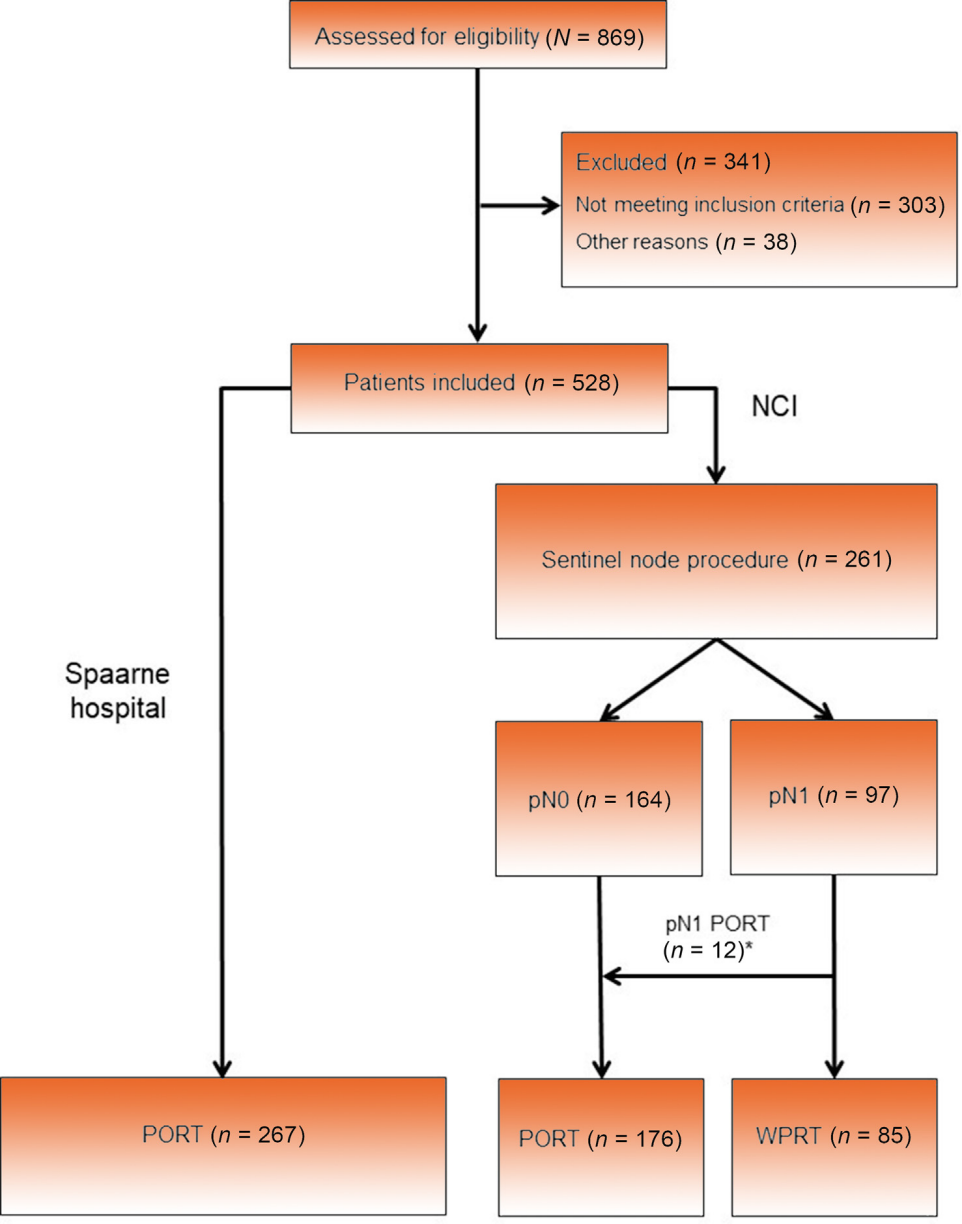
CONSORT diagram. NCI = Netherlands Cancer Institute; PORT = prostate-only radiotherapy; WPRT = whole pelvic radiotherapy. *Twelve patients with pN1 disease received PORT instead of WPRT because of a very small tumor deposit (*n* = 2), extensive lymph node dissection (*n* = 4), relative contraindication for WPRT (*n* = 2), or other reasons (*n* = 4).

A total of 528 eligible patients were retrospectively included (Fig. 1 and Table 1). Nodal staging was performed only radiologically in 267 patients (non-SLNB group) and with SLNB in 261 patients (SLNB group). The median follow-up length was 71 mo (95% confidence interval [CI] 66–76 mo): 76 mo (95% CI 70–82 mo) for the SLNB group and 66 mo (95% CI 59–73 mo) for the non-SLNB group. Compared with the non-SLNB group, patients in the SLNB group had a lower median age at diagnosis (65 vs 71 yr, *p* < 0.001), a higher rate of ≥cT3 tumors (62.8% vs 34.1%, *p* < 0.001), and a higher median Briganti nomogram–assessed risk of nodal metastases (42.7% vs 26.6%, *p* = 0.001). In the SLNB group, occult nodal metastases were found in 97 patients (37%) with a median metastasis size of 2 mm (interquartile range 1–4 mm). A total of 108 (14.7%) SNs were located outside the ePLND template (Supplementary Table 2), of which 45 (41.7%) were removed (in the remaining cases with both intra- and extratemplate SNs on preoperative LN mapping, we only removed SNs within the ePLND template).

**Table 1:** Patient and treatment characteristics stratified by nodal staging with or without an SLNB

	Overall (*n* = 528)	SLNB group (*n* = 261)	Non-SLNB group (*n* = 267)	*p* value
Age, median (IQR)	68 (64–73)	65 (62–69)	71 (67–75)	<0.001
iPSA, median (IQR)	15.5 (9.2–30.8)	14 (8.9–28.5)	16.9 (9.6–33.8)	0.03
T stage, *n* (%)				<0.001
T1c	86 (16.3)	28 (10.7)	58 (21.7)	
T2	187 (35.4)	69 (26.4)	118 (44.2)	
T3	237 (44.9)	153 (58.6)	84 (31.5)	
T4	18 (3.4)	11 (4.2)	7 (2.6)	
Radiological T stage, *n* (%)				<0.001
mT1	10 (1.9)	6 (2.3)	4 (1.5)	
mT2	105 (19.9)	56 (21.5)	49 (18.4)	
mT3	262 (49.6)	150 (57.5)	112 (41.9)	
mT4	10 (1.9)	8 (3.1)	2 (0.7)	
No radiological T stage, *n* (%)	141 (26.7)	41 (15.7)	100 (37.5)	
Pathological N stage, *n* (%)				NA
N0	NA	164 (63)	NA	
N1	NA	97 (37)	NA	
Radiological staging method, *n* (%)				0.001
PSMA PET	40 (7.5)	31 (11.9)	9 (3.4)	
Conventional imaging	475 (90)	224 (85.8)	251 (94)	
None	13 (2.5)	6 (2.3)	7 (2.6)	
ISUP grade group, *n* (%)				0.55
1	42 (8)	22 (8.4)	20 (7.5)	
2	143 (27.1)	71 (27.2)	72 (27)	
3	90 (17)	38 (14.6)	52 (19.5)	
4	152 (28.8)	75 (28.7)	77 (28.8)	
5	101 (19.1)	55 (21.1)	46 (17.2)	
EAU risk group, *n* (%)				<0.001
Low	2 (0.4)	1 (0.4)	1 (0.4)	
Intermediate	74 (14)	27 (10.3)	47 (17.6)	
High	197 (37.3)	69 (26.4)	128 (47.9)	
Locally advanced	255 (48.3)	164 (62.8)	91 (34.1)	
% Risk of LNM, median (IQR)	36.1 (15–62.1)	42.7 (17.4–67.8)	26.6 (13.4–54.3)	0.001
Number of sentinel nodes removed, median (IQR)	NA	2 (2–3)	NA	NA
Metastasis size (mm), median (IQR)	NA	2 (1–4)	NA	NA
Surgical duration (min), median (IQR)	NA	93 (75–115)	NA	NA
Hormonal treatment, *n* (%)				0.45
No ADT	16 (3)	6 (2.3)	10 (3.7)	
ADT	509 (96.4)	255 (97.7)	254 (95.1)	
Missing data	3 (0.6)	0 (0)	3 (1.1)	
ADT duration (mo), median (IQR)	36 (6–36)	36 (6–36)	31 (6–36)	0.48
Prostate radiation dose (Gy), median (IQR)	77 (77–78)	77 (75–77)	77 (77–78)	0.006
Pelvic radiation dose (Gy), *n* (%)				NA
50	NA	7 (2.7)		
52.5	NA	49 (18.8)		
56	NA	24 (9.2)		
Missing data	NA	10 (3.8)		
Radiotherapy fractions, median (IQR)	35 (35–38)	35 (35–38)	35 (35–39)	0.79
Radiation dose to anal sphincter (Gy), median (IQR)	15.4 (7.6–24.8)	17.5 (10.4–25.9)	14.1 (6.7–23.1)	0.003

ADT = androgen deprivation therapy; EAU = European Association of Urology; IQR = interquartile range; iPSA = initial prostate-specific antigen; ISUP = International Society of Urological Pathology; LNM = lymph node metastasis; NA = not applicable; PET = positron emission tomography; PSMA = prostate-specific membrane antigen; SLNB = sentinel lymph node biopsy.

### 3.1 BCR-free survival

**Fig. 2:**
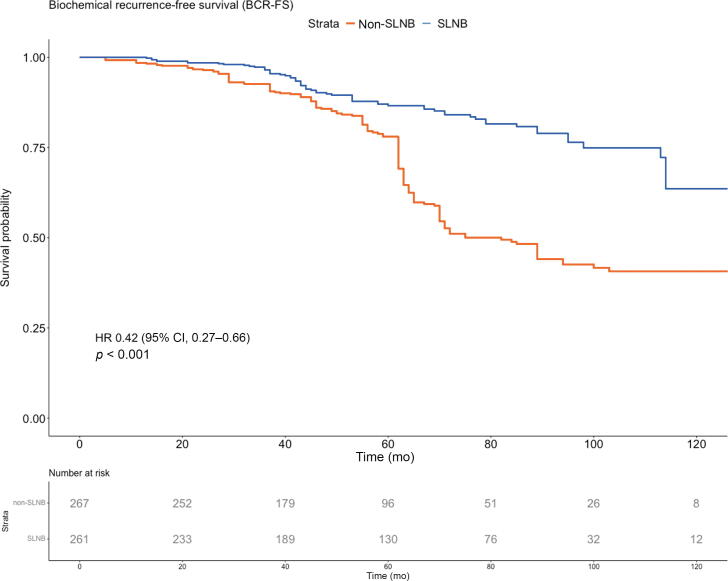

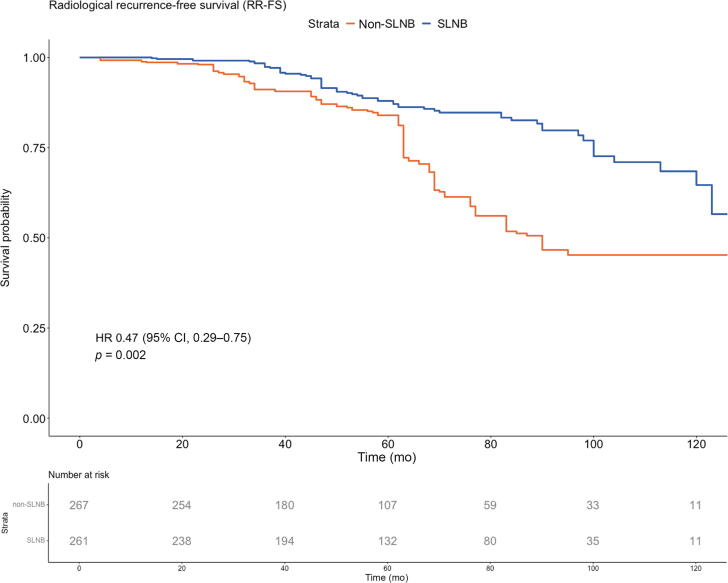

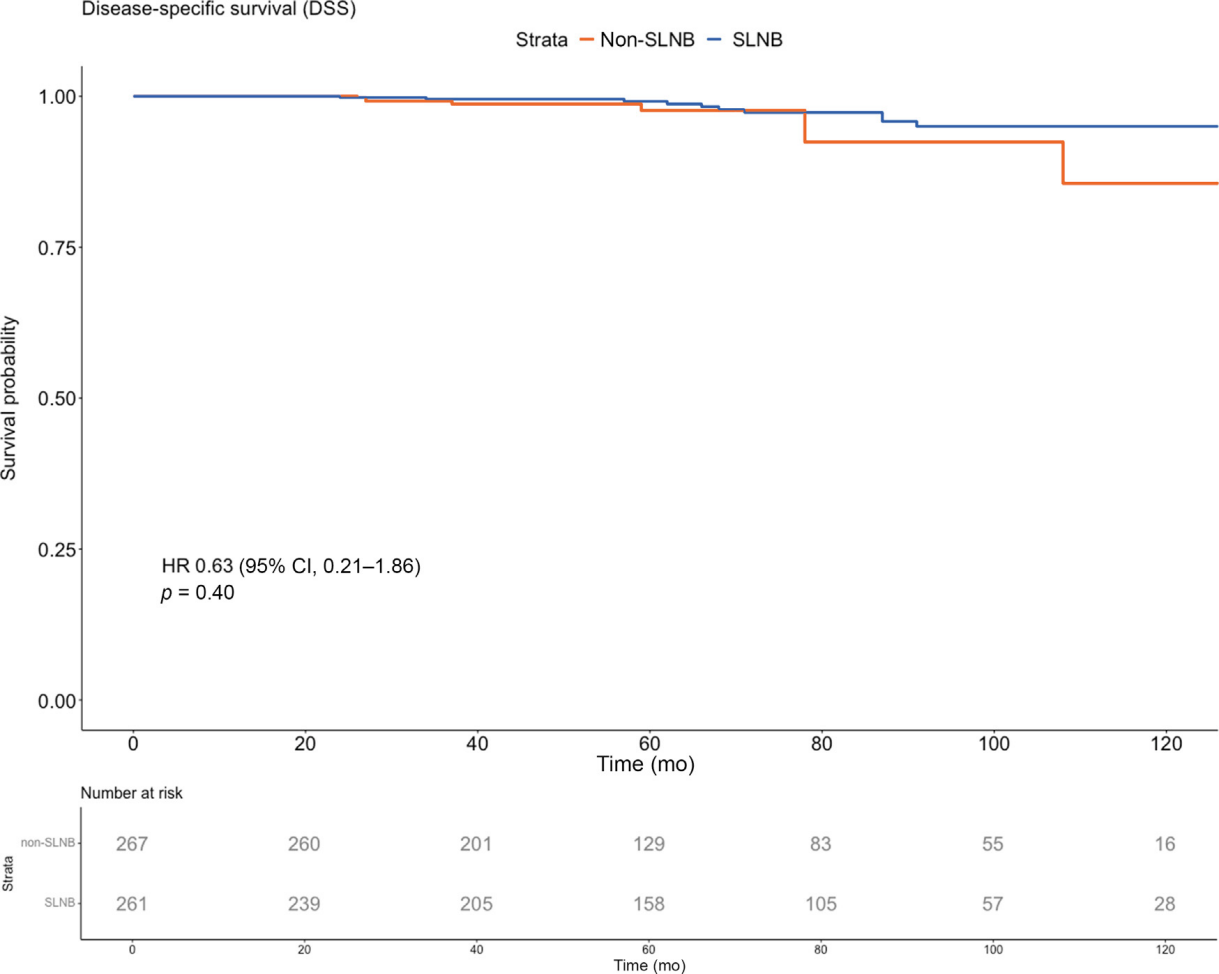
Adjusted and PSW Kaplan-Meier curves and numbers at risk for (A) biochemical recurrence–free survival, (B) radiological recurrence–free survival, and (C) disease-specific survival by treatment group. CI = confidence interval; HR = hazard ratio; PSW = propensity score weighted; SLNB = sentinel lymph node biopsy.

**Supplementary figure 1:**
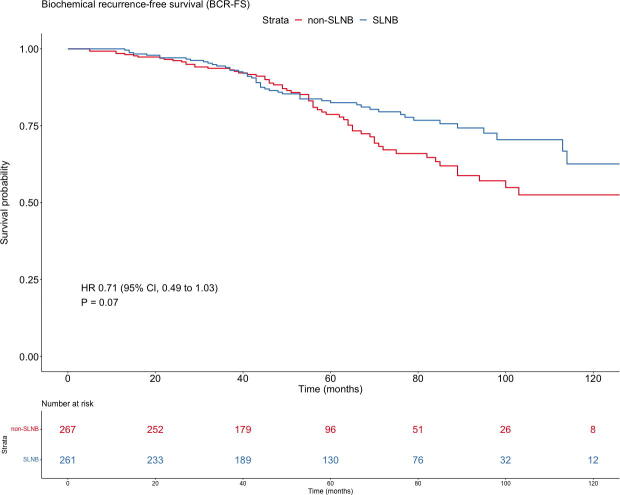


A covariate-adjusted PSW Kaplan-Meier curve for BCRFS is presented in Figure 2A (the unadjusted curve is presented in Supplementary Fig. 1A). Overall, 112 patients (21.2%) experienced a biochemical failure. The adjusted 5-, 6-, and 7-yr BCRFS rates were 87% (95% CI 83–90%), 84% (95% CI 80–88%), and 81% (95% CI 77–86%) in the SLNB group, and 78% (95% CI 74–83%), 51% (95% CI 45–58%), and 49% (95% CI 43–56%) in the non-SLNB group, respectively (*p* < 0.001). On PSW Cox multivariable regression analysis (Table 2; unweighted analysis is presented in Supplementary Table 3), SLNB was associated with significantly improved BCRFS (hazard ratio [HR] 0.38, 95% CI 0.25–0.59, *p* < 0.001), while clinical stage ≥cT3 (HR 2.35, 95% CI 1.53–3.63, *p* < 0.001) and ISUP grade ≥3 (HR 1.79, 95% CI 1.12–2.86, *p* = 0.015) were adverse prognostic factors.

**Table 2:** Propensity score weighted Cox regression analysis for biochemical recurrence, radiological recurrence, and disease-specific death (*n* = 528)

Predictor	Biochemical recurrence			Radiological recurrence			Disease-specific death		
	HR	95% CI	*p* value	HR	95% CI	*p* value	HR	95% CI	*p* value
*Univariable analysis*									
Group									
Non-SLNB	Ref	–	–	Ref	–	–	Ref	–	–
SLNB	0.42	0.27–0.66	<0.001	0.47	0.29–0.75	0.002	0.63	0.21–1.86	0.40
cT stage									
cT1-T2	Ref	–	–	Ref	–	–	Ref	–	–
cT3-T4	2.18	1.39–3.42	<0.001	2.62	1.59–4.30	<0.001	4.04	1.02–15.98	0.047
Log_2_iPSA	1.11	0.86–1.42	0.44	1.11	0.84–1.47	0.47	0.96	0.62–1.50	0.85
ISUP grade group									
1–2	Ref	–	–	Ref	–	–	Ref	–	–
3–5	1.77	1.11–2.83	0.02	2.22	1.33–3.68	0.002	2.57	0.81–8.13	0.11
ADT duration	1.01	0.99–1.02	0.5	1.01	1.00–1.03	0.08	1.03	1.01–1.05	0.008
Salvage treatment	–	–	–	–	–	–	16.58	4.45–61.73	<0.001
*Multivariable analysis*									
Group									
Non-SLNB	Ref	–	–	Ref	–	–	–	–	–
SLNB	0.38	0.25–0.59	<0.001	0.44	0.28–0.69	<0.001	–	–	–
cT stage									
cT1-T2	Ref	–	–	Ref	–	–	–	–	–
cT3-T4	2.35	1.53–3.63	<0.001	2.66	1.64–4.30	<0.001	–	–	–
Log_2_iPSA	1.16	0.95–1.42	0.14	1.15	0.94–1.41	0.18	–	–	–
ISUP grade group									
1–2	Ref	–	–	Ref	–	–	–	–	–
3–5	1.79	1.12–2.86	0.015	1.97	1.19–3.24	0.008	–	–	–
ADT duration	0.99	0.97–1.00	0.14	1.00	0.98–1.01	0.59	–	–	–

ADT = androgen deprivation therapy; CI = confidence interval; HR = hazard ratio; ISUP = International Society of Urological Pathology; iPSA = initial prostate-specific antigen; Ref = reference; SLNB = sentinel lymph node biopsy.

### 3.2 Radiological recurrence–free survival

Adjusted PSW Kaplan-Meier curves for RRFS are presented in Figure 2B (the unadjusted curve is presented in Supplementary Fig. 1B). A total of 98 (18.6%) patients experienced a radiological recurrence. Radiological recurrences were detected using PSMA PET/CT (57.7%), choline PET/CT (28.9%), or conventional imaging (13.4%). The distribution of imaging modalities was well balanced between the cohorts. The adjusted 5-, 6-, and 7-yr RRFS rates were, respectively, 88% (95% CI 84–92%), 85% (95% CI 80–90%), and 83% (95% CI 78–87%) in the SLNB group versus 84% (95% CI 80–88%), 61% (95% CI 56–68%), and 52% (95% CI 46-59%) in the non-SLNB group (*p* = 0.002). On PSW Cox multivariable regression analysis (Table 2; unweighted analysis is presented in Supplementary Table 3), SLNB was associated with significantly improved RRFS (HR 0.44, 95% CI 0.28–0.69, *p* < 0.001), while clinical stage ≥cT3 (HR 2.66, 95% CI 1.64–4.30, *p* < 0.001) and ISUP grade ≥ 3 (HR 1.97, 95% CI 1.19–3.24, *p* = 0.008) were adverse prognostic factors.

### 3.3 Disease-specific survival

Adjusted PSW Kaplan-Meier curves for DSS are demonstrated in Figure 2C (unadjusted curve is presented in Supplementary Fig. 1C). PCa-related death occurred in 20 patients (3.8%). The adjusted 5-, 6-, and 7-yr DSS rates were, respectively, 99% (95% CI 98–100%), 97% (95% CI 95–99%), and 97% (95% CI 95–99%) in the SLNB group versus 98% (95% CI 96–99%), 98% (95% CI 96–99%), and 92% (95% CI 89–96%) in the non-SLNB group (*p* = 0.40). On PSW Cox univariable analysis (Table 2; unweighted analysis is presented in Supplementary Table 3), clinical stage ≥cT3 (HR 4.04, 95% CI 1.02–15.98, *p* = 0.047) and ADT duration (HR 1.03, 95% CI 1.01–1.05, *p* = 0.008) showed a statistically significant association with DSS.

### 3.4 Complications and toxicity

The 90-d complication rates of the SLNB procedure are reported in Table 3. High-grade complications (Clavien-Dindo≥3) occurred in 11 patients (4.2%). No grade ≥4 complication was observed.

**Table 3:** Clavien-Dindo 90-d complications after sentinel lymph node biopsy

Clavien-Dindo grade	Complication	Patients, *n* (%)
1	Obturator nerve–related weakness or pain	8
	Ileus	2
	Collapse	1
	Diverticulitis	1
	Total	12 (4.6)
2	Urinary tract infection	17
	Fever of unknown etiology	9
	Wound infection	6
	Hematuria	1
	Pneumonia	1
	Total	34 (13)
3a	Infected lymphocele	2
	Urinary retention	1
	Total	3 (1.1)
3b	Perioperative bleeding with conversion to open surgery	2
	Postoperative bleeding	2
	Incarcerated umbilical hernia	1
	Abscess	1
	Ureteral injury	1
	Bladder perforation	1
	Total	8 (3.1)

Overall, 64 (12.1%) and 159 (30.1%) patients experienced grade 2 or 3 gastrointestinal (GI) or genitourinary (GU) toxicity, respectively (Table 4). No grade 4 or 5 toxicities were observed. Patients receiving WPRT had significantly higher GI and GU toxicity rates than those receiving PORT. Compared with the non-SLNB group, more patients in the SN-directed PORT arm experienced mild-to-moderate GI (18.3% vs 1.9%) and GU (39.8% vs 16.6%) toxicities, but no statistically significant difference was observed in the overall grade 3 toxicity.

**Table 4:** Treatment-associated toxicities, CTCAE V5.0 grade ≥2

Complication	All patients (*n* = 528), *n* (%)	PORT without SN (*n* = 267), *n* (%)	PORT with SN (*n* = 176), *n* (%)	WPRT (*n* = 85), *n* (%)	*p* value
*Gastrointestinal toxicity*					
Constipation					<0.001
Grade 2	2 (0.4)	0 (0)	1 (0.6)	1 (1.2)	
Diarrhea					
Grade 2	21 (4)	0 (0)	5 (2.8)	16 (18.8)	
Grade 3	1 (0.2)	0 (0)	0 (0)	1 (1.2)	
Proctitis					
Grade 2	45 (8.5)	5 (1.9)	26 (14.8)	14 (16.5)	
Grade 3	14 (2.7)	2 (0.7)	8 (4.5)	4 (4.7)	
Overall grade ≥2 gastrointestinal toxicity	64 (12.1)	5 (1.9)	31 (18.3)	28 (32.9)	<0.001
*Genitourinary toxicity*					
Urinary tract infection					<0.001
Grade 2	29 (5.5)	11 (4.1)	11 (6.3)	7 (8.2)	
Grade 3	3 (0.6)	1 (0.4)	0 (0)	2 (2.4)	
Hematuria					
Grade 2	6 (1.1)	1 (0.4)	1 (0.6)	4 (4.7)	
Urinary urgency and/or frequency					
Grade 2	61 (11.6)	0 (0)	39 (22.2)	22 (25.9)	
Grade 3	2 (0.4)	2 (0.7)	0 (0)	0 (0)	
Urinary retention					
Grade 2	115 (21.8)	33 (12.4)	53 (30.1)	29 (34.1)	
Erectile dysfunction					0.017
Grade 2	38 (7.2)	7 (2.6)	19 (10.8)	12 (14.1)	
Grade 3	6 (1.1)	5 (1.9)	0 (0)	1 (1.2)	
Fatigue					1.00
Grade 2	7 (1.3)	2 (0.7)	4 (2.3)	1 (1.2)	
Hot flashes					0.395
Grade 2	43 (8.1)	17 (6.4)	17 (9.7)	9 (10.6)	
Grade 3	1 (0.2)	1 (0.4)	0 (0)	0 (0)	
Overall grade ≥2 genitourinary toxicity	159 (30.1)	44 (16.6)	70 (39.8)	45 (52.9)	<0.001
Overall grade 3 toxicity	27 (5.1)	11 (4.1)	8 (4.5)	8 (9.4)	0.060

CTCAE = Common Terminology Criteria for Adverse Events; PORT = prostate-only radiotherapy; SN = sentinel node; WPRT = whole-pelvis radiotherapy.

### 3.5 Patterns of radiological recurrence

Patterns of radiological recurrence are demonstrated in Supplementary Table 4. In the overall cohort, local recurrence was most common (43 patients, 8.1%, biopsy proven in 18 patients [41.9%]), followed by regional LNMs (39 patients, 7.4%) and bone metastases (31 patients, 5.9%). A regional LNM was observed in ten patients (3.8%) in the SLNB group (two patients treated with WPRT and eight patients treated with PORT) and 29 patients (10.9%) in the non-SLNB group. The regional LN recurrences in two patients treated with WPRT occurred outside the WPRT field (ie, outside the region that received a high or an elective dose).

## 4 Discussion

To our knowledge, this is the first PSW study that demonstrates favorable oncological outcomes for SLNB-based selection of histologically node-positive PCa patients for WPRT. These findings were compared with (conventional) imaging-directed PORT in cN0 patients with an increased risk of nodal metastases. When corrected for baseline characteristics, SLNB-guided radiotherapy was associated with improved BCRFS and RRFS. These improved oncological outcomes could be attributed to the additional pelvic irradiation and the longer course of ADT for pN1 patients. It can be assumed that patients in the non-SLNB group with clinically occult nodal metastases would also benefit from a similar therapeutic approach. The difference in 5- and 7-yr survival in the PORT group may be explained by the testosterone recovery period after ADT. A testosterone recovery period of >2 yr after hormone therapy has been reported [22], and a longer duration of hormone therapy and older age are significantly associated with a prolonged recovery interval [23]. It is plausible that testosterone levels were still recovering 5 yr after the start of radiotherapy, resulting in a later onset of recurrences.

Our initial efforts to document the oncological outcomes of SLNB-guided radiotherapy were the results of a single-arm study that compared the outcomes of SLNB-guided radiotherapy versus Kattan nomogram–predicted BCR rates [24]. The results in the present study come from an extended SLNB cohort (including patients up until 2018 instead of 2016) with longer follow-up (71 vs 52 mo). Here, we found an adjusted 5-yr BCRFS rate of 81.9% in 85 pN1 patients who received WPRT. Previous literature on WPRT in pN1 patients using ePLND as a staging tool has shown a 5-yr BCRFS rate of 65–67% [3], [4]. Hence, application of WPRT in pN1 patients staged using SLNB procedures provides favorable results, while omitting the morbidity associated with ePLND [5].

Randomized trials (ie, RTOG 9413 and GETUG-01) on prophylactic elective WPRT failed to show a survival benefit for WPRT compared with PORT [25], [26]. However, the more recent POP-RT trial including (very) high-risk cN0 PCa patients has shown BCRFS and disease-free survival benefits of WPRT combined with long-term use of ADT [5]. As such, it could be that the inclusion of patients with a low risk of nodal metastases, short ADT duration, and relatively low radiation doses has diluted the benefit of WPRT in earlier trials. The unadjusted survival rates of our non-SLNB cohort are comparable with the outcomes of the PORT group in the POP-RT trial. However, the fact that the 5-yr BCRFS (95%) of the WPRT group in the POP-RT study far exceeded the outcomes of WPRT (82%), not only in our population, but also in WPRT populations in the literature (65–67%) [3], [4], can be explained by three facts. First, lifetime androgen deprivation was achieved by surgical castration in 14.5% of WPRT patients in the POP-RT trial. Second, PSMA PET staging was performed in 80% of the POP-RT patients. Third, the WPRT population in the POP-RT trial included only cN0 patients, whereas the WPRT patients in our and the aforementioned studies included only pN1 patients.

Our grade 2 toxicity rates are higher than those reported elsewhere [6], [7], [8]. The use of different toxicity grading systems and variability in the documentation, interpretation, and scoring of toxicity may explain this difference. The POP-RT study reports significantly higher late GU toxicity (grade ≥2) for elective WPRT (17.7%) than PORT (7.5%, *p* = 0.03) [5]. Higher GI or GU toxicity rates after WPRT in both our study and previous literature stress the importance of adequate patient selection [6], [7], [8]. In that sense, our approach helped select patients with a pathologically negative SN (63%) for PORT as treatment rather than WPRT. Although mild-to-moderate GI and GU toxicity was higher in the SLNB-directed PORT arm than in the non-SLNB group—surgery in the small pelvis may contribute to increased toxicity, overall high-grade toxicity did not differ between the two arms. We believe that the high-grade complication rate of 4% of the SLNB procedure justifies its use to select only pN1 patients for WPRT, and to avoid pelvic irradiation and its toxicity in pN0 patients.

An important observation was that in the current study, none of the patients treated with WPRT had an LN recurrence inside the radiotherapy field. In line with previous studies, this suggests that WPRT is effective in preventing in-field nodal recurrences [4], [27]. Moreover, regional LN recurrence rates were lower in the non-SLNB group than in the SLNB group (4.5% vs 10.9%). It is plausible that a subset of non-SLNB patients had clinically occult pelvic nodal metastases at primary staging that were detected during follow-up, resulting in a higher rate of regional nodal recurrences.

In our cohort, the surgical SLNB procedure had an overall 90-d complication rate of 21.8%. This is markedly lower than the up to 51% complication rate reported for ePLND [12], but higher than the 9% complication rate reported previously for SLNB procedures [24]. However, when comparing the rate of severe complications (Clavien-Dindo grade ≥3), the rate of 5% reported previously for SLNB procedures [24] is comparable with the rate of 4.2% observed in our cohort.

One of the main limitations of our study is the bias inherent to its retrospective design in addition to the missing data and loss of follow-up, since many patients went back to their referring hospital following treatment. In addition, all patients were treated in two hospitals that share a radiotherapy facility, which might have introduced a center effect bias. Since imaging to detect radiological recurrences was performed as part of routine clinical care, the scans may have been performed at different time points, resulting in inconsistent imaging intervals. We attempted to control for bias with PSW analyses. Unfortunately, PSW analyses cannot exclude a selection bias or bias by unknown variables, and hence cannot replace a randomized controlled trial. The use of different tracers for the SLNB procedure introduced heterogeneity into our study. However, the pN1 rate did not differ significantly between patients who received SLNB with Tc-nanocolloid and those who received it with ICG-Tc-nanocolloid (36.2% vs 37.8%, *p* = 0.897). Some patients in our cohort were also included in (ongoing) radiotherapy trials and therefore received nonstandard (hypofractionated) radiotherapy dosing schemes (*n* = 60; 11.4%). For various reasons, 12 out 97 (12.4%) pN1 patients received PORT instead of WPRT. It should be noted that higher BCR and radiological recurrence rates in pN1 patients treated with PORT than in those treated with WPRT suggest a benefit from WPRT in this population. In addition, the diagnostic value of SLNB in PCa has yet to be validated in a randomized trial. High diagnostic accuracy is necessary to distinguish between pN0 and pN1 patients. The high sensitivity and negative predictive value of SLNB [15], combined with the low rate of regional nodal recurrences (4.5%) in our SLNB pN0 population, suggest that the majority of pN0 SLNB patients were truly node negative. Assuming that scoring more severe toxicities would overcome the limitations of retrospective data collection, we scored only grade ≥2 toxicities. Lastly, given the period of inclusion, the majority of the patients were staged using conventional imaging methods and only 40 patients (7.5%) were staged primarily using PSMA PET. Of these patients, 31 underwent SLNB and 12 (38.7%) had pN1 disease. As the median metastasis size of 2 mm in our cohort lies below the reported 3 mm detection limit of PSMA PET and PSMA-based intraoperative radioguidance techniques [28], [29], [30], it is questionable whether the use of PSMA PET or intraoperative PSMA radioguidance would have impacted the nodal detection rate and treatment allocation given the low sensitivity of PSMA PET for nodal metastases <3 mm [10], [11]. The diagnostic value of the SLNB procedure in PCa patients with localized disease on PSMA PET/CT will be evaluated in a future study.

## 5 Conclusions

In conclusion, SLNB-based selection of pN1 patients for WPRT is associated with favorable oncological outcomes as compared with imaging-based PORT in cN0 PCa patients. The safety profile of this treatment option is acceptable with a low rate of high-grade complications. By applying SLNB procedures as a means to select pN1 patients, the use of WPRT could be limited to patients that actually benefitted from the procedure. The lack of clear guideline recommendations on the use of WPRT in primary PCa [2] and the overall promising results from this study can be valid arguments for a randomized controlled trial comparing SLNB-guided radiotherapy versus imaging-based radiotherapy.

  ***Author contributions*:** Hilda A. de Barros had full access to all the data in the study and takes responsibility for the integrity of the data and the accuracy of the data analysis.

  *Study concept and design*: de Barros, P.J. van Leeuwen, van der Poel.

*Acquisition of data*: de Barros, Duin, Mulder, Noordzij, Wit, Pos, Vogel, Schaake, P.J. van Leeuwen, F.W.B. van Leeuwen, van der Poel.

*Analysis and interpretation of data*: de Barros, Duin, Mulder, van der Noort, Schaake, P.J. van Leeuwen, van der Poel.

*Drafting of the manuscript*: de Barros, Duin, Mulder, Grivas, P.J. van Leeuwen, van der Poel.

*Critical revision of the manuscript for important intellectual content*: Duin, Mulder, van der Noort, Noordzij, Wit, Pos, Vogel, Schaake, F.W.B. van Leeuwen, P.J. van Leeuwen, Grivas, van der Poel.

*Statistical analysis*: de Barros, Duin, van der Noort.

*Obtaining funding*: None.

*Administrative, technical, or material support*: None.

*Supervision*: Schaake, P.J. van Leeuwen, van der Poel.

*Other*: None.

  ***Financial disclosures:*** Hilda A. de Barros certifies that all conflicts of interest, including specific financial interests and relationships and affiliations relevant to the subject matter or materials discussed in the manuscript (eg, employment/affiliation, grants or funding, consultancies, honoraria, stock ownership or options, expert testimony, royalties, or patents filed, received, or pending), are the following: None.

  ***Funding/Support and role of the sponsor*:** None.

## Figures and Tables

**Supplementary figure 2:**
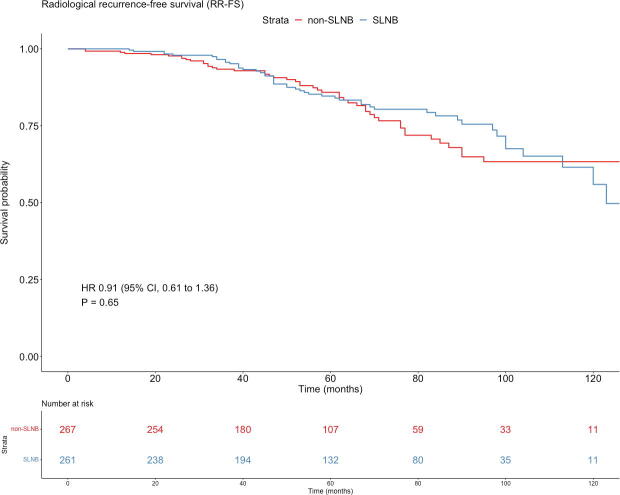


**Supplementary figure 3:**
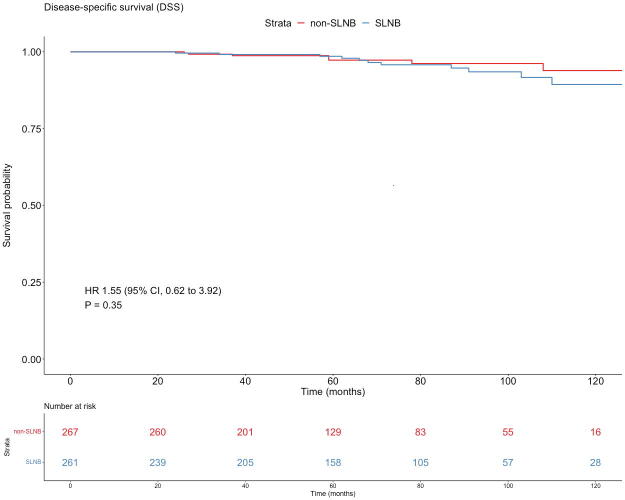


## References

[1] Bernstein A.N., Shoag J.E., Golan R. (2018). Contemporary incidence and outcomes of prostate cancer lymph node metastases. J Urol.

[2] Mottet N., van den Bergh R.C.N., Briers E. (2021). EAU-EANM-ESTRO-ESUR-SIOG guidelines on prostate cancer—2020 update. Part 1: screening, diagnosis, and local treatment with curative intent. Eur Urol.

[3] Van Hemelryk A., De Meerleer G., Ost P. (2016). The outcome for patients with pathologic node-positive prostate cancer treated with intensity modulated radiation therapy and androgen deprivation therapy: a case-matched analysis of pN1 and pN0 patients. Int J Radiat Oncol.

[4] Poelaert F., Fonteyne V., Ost P. (2017). Whole pelvis radiotherapy for pathological node-positive prostate cancer. Strahlentherapie Und Onkol.

[5] Murthy V., Maitre P., Kannan S. (2021). Prostate-only versus whole-pelvic radiation therapy in high-risk and very high-risk prostate cancer (POP-RT): outcomes from phase III randomized controlled trial. J Clin Oncol.

[6] Murthy V., Maitre P., Bhatia J. (2020). Late toxicity and quality of life with prostate only or whole pelvic radiation therapy in high risk prostate cancer (POP-RT): a randomised trial. Radiother Oncol.

[7] Tharmalingam H, Tsang Y, Choudhury A, Hoskin P. External beam radiotherapy (EBRT) and high dose rate (HDR) brachytherapy for intermediate and high-risk prostate cancer: whole pelvis or prostate-only EBRT? J Clin Oncol 2018;36:86–86.

[8] Roach M., Moughan J., Lawton C.A.F. (2018). Sequence of hormonal therapy and radiotherapy field size in unfavourable, localised prostate cancer (NRG/RTOG 9413): long-term results of a randomised, phase 3 trial. Lancet Oncol.

[9] Briganti A., Abdollah F., Nini A. (2012). Performance characteristics of computed tomography in detecting lymph node metastases in contemporary patients with prostate cancer treated with extended pelvic lymph node dissection. Eur Urol.

[10] Perera M., Papa N., Roberts M. (2020). Gallium-68 prostate-specific membrane antigen positron emission tomography in advanced prostate cancer—updated diagnostic utility, sensitivity, specificity, and distribution of prostate-specific membrane antigen-avid lesions: a systematic review and meta-analysis. Eur Urol.

[11] Hope T.A., Eiber M., Armstrong W.R. (2021). Diagnostic accuracy of 68 Ga-PSMA-11 PET for pelvic nodal metastasis detection prior to radical prostatectomy and pelvic lymph node dissection. JAMA Oncol.

[12] Briganti A., Blute M.L., Eastham J.H. (2009). Pelvic lymph node dissection in prostate cancer. Eur Urol.

[13] Joniau S., Van den Bergh L., Lerut E. (2013). Mapping of pelvic lymph node metastases in prostate cancer. Eur Urol.

[14] van der Poel H.G., Wit E.M., Acar C. (2017). Sentinel node biopsy for prostate cancer: report from a consensus panel meeting. BJU Int.

[15] Wit E.M.K., Acar C., Grivas N. (2017). Sentinel node procedure in prostate cancer: a systematic review to assess diagnostic accuracy. Eur Urol.

[16] Mattei A., Fuechsel F.G., Bhatta Dhar N. (2008). The template of the primary lymphatic landing sites of the prostate should be revisited: results of a multimodality mapping study. Eur Urol.

[17] Briganti A., Larcher A., Abdollah F. (2012). Updated nomogram predicting lymph node invasion in patients with prostate cancer undergoing extended pelvic lymph node dissection: the essential importance of percentage of positive cores. Eur Urol.

[18] van der Poel H.G., Buckle T., Brouwer O.R., Valdés Olmos R.A., van Leeuwen F.W.B. (2011). Intraoperative laparoscopic fluorescence guidance to the sentinel lymph node in prostate cancer patients: clinical proof of concept of an integrated functional imaging approach using a multimodal tracer. Eur Urol.

[19] Lawton C.A.F., Michalski J., El-Naqa I. (2009). RTOG GU radiation oncology specialists reach consensus on pelvic lymph node volumes for high-risk prostate cancer. Int J Radiat Oncol Biol Phys.

[20] Roach M., Hanks G., Thames H. (2006). Defining biochemical failure following radiotherapy with or without hormonal therapy in men with clinically localized prostate cancer: recommendations of the RTOG-ASTRO Phoenix Consensus Conference. Int J Radiat Oncol Biol Phys.

[21] Cole S.R., Hernán M.A. (2008). Constructing inverse probability weights for marginal structural models. Am J Epidemiol.

[22] D’Amico A.V., Chen M.H., Renshaw A.A., Loffredo M., Kantoff P.W. (2009). Interval to testosterone recovery after hormonal therapy for prostate cancer and risk of death. Int J Radiat Oncol Biol Phys.

[23] Nam W., Choi S.Y., Yoo S.J. (2018). Factors associated with testosterone recovery after androgen deprivation therapy in patients with prostate cancer. Investig Clin Urol.

[24] Grivas N., Wit E., Pos F. (2017). Sentinel lymph node dissection to select clinically node-negative prostate cancer patients for pelvic radiation therapy: effect on biochemical recurrence and systemic progression. Int J Radiat Oncol.

[25] Pommier P., Chabaud S., Lagrange J.L. (2007). Is there a role for pelvic irradiation in localized prostate adenocarcinoma? Preliminary results of GETUG-01. J Clin Oncol.

[26] Roach M., DeSilvio M., Lawton C. (2003). Phase III trial comparing whole-pelvic versus prostate-only radiotherapy and neoadjuvant versus adjuvant combined androgen suppression: Radiation Therapy Oncology Group 9413. J Clin Oncol.

[27] Liskamp C.P., Donswijk M.L., van der Poel H.G., Schaake E.E., Vogel W.V. (2020). Nodal recurrence patterns on PET/CT after RTOG-based nodal radiotherapy for prostate cancer. Clin Transl Radiat Oncol.

[28] Gandaglia G., Mazzone E., Stabile A. (2022). Prostate-specific membrane antigen radioguided surgery to detect nodal metastases in primary prostate cancer patients undergoing robot-assisted radical prostatectomy and extended pelvic lymph node dissection: results of a planned interim analysis of a prospective phase 2 study. Eur Urol.

[29] Maurer T., Robu S., Schottelius M. (2019). 99mTechnetium-based prostate-specific membrane antigen–radioguided surgery in recurrent prostate cancer. Eur Urol.

[30] de Barros H.A., van Oosterom M.N., Donswijk M.L. (2022). Robot-assisted prostate-specific membrane antigen–radioguided salvage surgery in recurrent prostate cancer using a DROP-IN gamma probe: the first prospective feasibility study. Eur Urol.

