# Supplementary material for: Sentinel Node Procedure to Select Clinically Localized Prostate Cancer Patients with Occult Nodal Metastases for Whole Pelvis Radiotherapy
Source: Eur Urol Open Sci. 2023 Jan 30;49:80–9. doi: 10.1016/j.euros.2022.12.011 (PMC9975002; doi:10.1016/j.euros.2022.12.011)
Supplement: Supplementary data 1 [file mmc1.docx]

**Supplementary File 1**

**Tracer administration and pre-operative SN mapping**

Either 99mTechnetium (Tc)-nanocolloid or ICG-99mTechnetium (Tc)-nanocolloid was injected transrectally under ultrasonic guidance into the peripheral zone in four quadrants of the prostate. An early and late lymphoscintigram (15 minutes and 2 hours after injection, respectively) was followed by a Single Photon Emission Computed Tomography (SPECT) and low-dose CT scan. 3D SPECT/CT reconstruction rendered anatomical localization of SNs pre-operatively. An experienced nuclear medicine physician interpreted the images, which served as a roadmap for the intraoperative localization of SNs.

**Supplementary Table 1 - Multivariable logistic regression predicting the propensity of receiving a sentinel node procedure**

| Predictor | HR | 95% CI | p value |
| --- | --- | --- | --- |
| Age | 0.83 | 0.80-0.86 | <0.001 |
| cT stage |  |  |  |
| cT1-T2 | Ref | - | - |
| cT3-T4 | 3.69 | 2.43-5.62 | <0.001 |
| Log_2_iPSA | 0.78 | 0.66 – 0.94 | 0.007 |
| ISUP grade group |  |  |  |
| 1-2 | Ref | - | - |
| 3-5 | 0.60 | 0.56-1.40 | 0.60 |
| ADT duration (months) | 1.00 | 0.98 – 1.01 | 0.52 |

**Supplementary Table 2 - Location of detected sentinel lymph nodes**

| **Location** | **Number of nodes (%)** |
| --- | --- |
| Obturator, n (%) | 252 (34.2%) |
| External iliac, n (%) | 108 (14.7%) |
| Internal Iliac, n (%) | 86 (11.7%) |
| Common iliac, n (%) | 183 (24.8%) |
| Paravesical, n (%) | 13 (1.8%) |
| Pararectal, n (%) | 22 (3.0%) |
| Presacral, n (%) | 42 (5.7%) |
| Para-aortal, n (%) | 17 (2.3%) |
| Inguinal, n (%) | 6 (0.8%) |
| Cloquet, n (%) | 6 (0.8%) |
| Musculus gluteus maximus, n (%) | 1 (0.1%) |
| Promontory, n (%) | 1 (0.1%) |

**Supplementary Table 3 - Cox regression analysis for biochemical recurrence, radiological recurrence and disease-specific death (n = 528)**

|  | Biochemical recurrence | | | Radiological recurrence | | | Disease-specific death | | |
| --- | --- | --- | --- | --- | --- | --- | --- | --- | --- |
| Predictor | HR | 95% CI | p value | HR | 95% CI | p value | HR | 95% CI | p value |
| Univariable analysis | | | | | | | | | |
| Group |  |  |  |  |  |  |  |  |  |
| Non-SLNB | Ref | - | - | Ref | - | - | Ref | - | - |
| SLNB | 0.71 | 0.49-1.03 | 0.073 | 0.91 | 0.61-1.36 | 0.65 | 1.55 | 0.62-3.92 | 0.35 |
| cT stage |  |  |  |  |  |  |  |  |  |
| cT1-T2 | Ref | - | - | Ref | - | - | Ref | - | - |
| cT3-T4 | 1.63 | 1.11-2.40 | 0.013 | 2.10 | 1.37-3.21 | <0.001 | 4.75 | 1.39-16.22 | 0.013 |
| Log_2_iPSA | 1.23 | 1.06-1.42 | 0.0053 | 1.23 | 1.08-1.48 | 0.003 | 1.28 | 0.92-1.79 | 0.15 |
| ISUP grade group |  |  |  |  |  |  |  |  |  |
| 1-2 | Ref | - | - | Ref | - | - | Ref | - | - |
| 3-5 | 1.32 | 0.89-1.96 | 0.17 | 1.62 | 1.05-2.51 | 0.031 | 1.45 | 0.55-3.78 | 0.45 |
| ADT duration | 1.00 | 0.99-1.01 | 0.83 | 1.01 | 1.00-1.02 | 0.10 | 1.03 | 1.00-1.05 | 0.047 |
| Salvage treatment | - | - | - | - | - | - | 11.70 | 3.36-40.76 | <0.001 |
| Multivariable analysis | | | | | | | | | |
| Group |  |  |  |  |  |  |  |  |  |
| Non-SLNB | Ref | - | - | Ref | - | - | - | - | - |
| SLNB | 0.55 | 0.37-0.82 | 0.004 | 0.69 | 0.45-1.07 | 0.10 | - | - | - |
| cT stage |  |  |  |  |  |  |  |  |  |
| cT1-T2 | Ref | - | - | Ref | - | - | - | - | - |
| cT3-T4 | 1.94 | 1.27-2.95 | 0.002 | 2.18 | 1.37-3.47 | <0.001 | - | - | - |
| Log_2_iPSA | 1.24 | 1.07-1.45 | 0.005 | 1.24 | 1.05-1.45 | 0.010 | - | - | - |
| ISUP grade group |  |  |  |  |  |  |  |  |  |
| 1-2 | Ref | - | - | Ref | - | - | - | - | - |
| 3-5 | 1.40 | 0.91-2.14 | 0.13 | 1.50 | 0.93-2.40 | 0.09 | - | - | - |
| ADT duration | 0.99 | 0.98-1.00 | 0.05 | 1.00 | 0.98-1.01 | 0.74 | - | - | - |

**Supplementary Table 4 - Radiological recurrence patterns stratified by treatment group.**

| Location, N (%) | All patients  (n = 528) | Non-SLNB  (n = 267) | SLNB-PORT  (n = 176) | SLNB-WPRT  (n = 85) |
| --- | --- | --- | --- | --- |
| Local | 43 (8.1) | 16 (6) | 13 (3.4) | 14 (16.5) |
| Node | 46 (8.7) | 33 (12.4) | 9 (5.1) | 4 (4.8) |
| Regional | 39 (7.4) | 29 (10.9) | 8 (4.5) | 2 (2.4) |
| Non-regional | 7 (1.3) | 4 (1.5) | 1 (0.6) | 2 (2.4) |
| Bone | 31 (5.9) | 16 (6) | 7 (4) | 8 (9.5) |
| Axial | 13 (2.5) | 9 (3.4) | 2 (1.1) | 2 (2.4) |
| Non-axial | 7 (1.3) | 1 (0.4) | 4 (2.3) | 2 (2.4) |
| Axial + non-axial | 11 (2.1) | 6 (2.2) | 1 (0.6) | 4 (4.7) |
| Visceral | 4 (0.8) | 0 (0) | 1 (0.6) | 3 (3.5) |

SLNB = sentinel lymph node biopsy; PORT = prostate-only radiotherapy; WPRT = whole-pelvis radiotherapy

**Supplementary Figure 1. Kaplan-Meier curves and numbers at risk for (A) biochemical recurrence-free survival, (B) radiological recurrence-free survival, and (C) disease-specific survival by treatment group.** CI = confidence interval; HR = hazard ratio; SLNB = sentinel lymph node biopsy
